# Trithorax group genes in hematopoiesis

**DOI:** 10.18632/oncotarget.4882

**Published:** 2015-07-19

**Authors:** Jennifer Chase, Jolanta Grembecka, Ivan Maillard

**Affiliations:** Center for Stem Cell Biology, Life Sciences Institute, Division of Hematology-Oncology, Department of Internal Medicine, Department of Cell and Developmental Biology, University of Michigan, Ann Arbor, MI, USA

Hematopoietic stem and progenitor cells maintain blood homeostasis by giving rise to all mature blood cells and lymphocytes. A recent study identified the trithorax group gene *ash1l* as a critical regulator of quiescence and self-renewal potential in adult hematopoietic stem cells [1]. *Ash1l* encodes a large protein with histone methyltransferase activity that cooperates with the leukemia-associated gene *Mll1* to regulate hematopoiesis in mice.

Epigenetic modifiers have essential functions in normal hematopoiesis and are frequently dysregulated in hematological malignancies. Trithorax group (TrxG) genes were discovered in *Drosophila melanogaster* for their role in inducing normal body patterning by positively regulating *Homeobox* (*Hox)* gene expression [2]. *Hox* genes also play critical roles in mammalian development and in specific adult tissues, including the hematopoietic system. Although multiple members of the TrxG gene network interact genetically in flies [3], much less is known about their crosstalk and cooperativity in mammals.

*Mixed Lineage Leukemia 1* (*MLL1*) is the mammalian homolog of fly *trithorax*. *MLL1* was originally discovered as a recurrent translocation partner in acute leukemias (reviewed in [4]). *MLL1* maintains expression of *HOX* genes during development and regulates the function of normal hematopoietic stem cells. MLL1-driven leukemias are characterized by upregulated expression of *HOXA* cluster genes. Endogenous MLL1 contains a Su(var)3-9, Enhancer-of-zeste and Trithorax (SET) domain with H3K4 histone methyltransferase (HMT) activity that is lost in MLL1 fusion proteins [4]. MLL1 functions as part of a multiprotein complex that includes RBP4, WDR5, ASH2L, and the cofactor menin. In contrast, MLL1 fusion proteins consist of the N-terminal domain of MLL1 fused to an oncogenic partner. This complex also associates with the cofactor menin, but lacks intrinsic H3K4 HMT activity, which can be provided by endogenous MLL1 encoded by the non-rearranged allele [4]. In addition, the H3K79 histone methyltransferase DOT1L is recruited to the oncogenic complex at least in part through interactions with the MLL1 fusion partners AF10, AF4, AF9 or ENL [4]. Multiple research groups are focusing on targeting members of this complex in leukemia. Careful consideration must be given to disrupting oncogenic transformation while maintaining normal hematopoiesis, for example by targeting regulatory features of the complex that are unique or dominant in malignant cells.

Besides *MLL1*, other TrxG members are poorly characterized in mammals. The TrxG gene *ash1* (*absent, small, or homeotic discs 1*) was discovered in flies and shown to interact genetically with other TrxG genes [3]. Its mammalian homolog *Ash1-like* (*Ash1l)* encodes a large protein that contains a SET domain with *in vitro* H3K36 histone methyltransferase activity [5]. We recently discovered that *Ash1l* is a critical regulator of adult hematopoietic stem cells (HSCs) [1]. *Ash1l*-deficient mice generate normal numbers of fetal and neonatal long-term HSCs, but these cells become rapidly depleted in young adult mice. Phenotypically defined *Ash1l*-deficient HSCs display decreased quiescence and fail to give rise to long-term tri-lineage hematopoietic output after transplantation to irradiated recipients, indicating profoundly defective function. Moreover, *Ash1l*-deficient HSCs compete poorly for niche space, as evidenced by potent engraftment of wild-type HSCs in *Ash1l*-deficient recipients even in the absence of irradiation. Despite these defects, *Ash1l*-deficient mice maintain steady-state hematopoiesis and display enhanced self-renewal of progenitors downstream of HSCs. As observed for *Mll1* in normal and malignant hematopoiesis, *Ash1l* regulates expression of *Hoxa* cluster genes [1]. Individual deficiency of *Ash1l* or *Mll1* results in decreased but not abolished *Hoxa* expression [1, 6]. Functionally, inactivation of both *Mll1* and *Ash1l* but not either gene alone leads to rapid hematopoietic failure [1]. Altogether, this is the first *in vivo* demonstration of cooperativity between mammalian TrxG proteins. More work is needed to define the critical pathways operating downstream of *Ash1l* and *Mll1* in HSCs and the molecular mechanisms of their cooperative effects.

As the field moves forward in targeting TrxG proteins in leukemia, careful consideration should be given to understanding how these proteins function during steady-state hematopoiesis in order to exploit critical structural and functional differences. For example, recent work showed that small molecular inhibitors of the menin/MLL1 interaction have substantial *in vivo* therapeutic activity in mouse models of MLL1-driven leukemia, but minimal negative impact on normal hematopoiesis [7]. Menin is an MLL1 cofactor that is essential for its recruitment to a subset of target genes [6] and is part of the MLL1 complex in normal hematopoiesis and leukemia. Combined deficiency of *Men1* and *Ash1l* in mice results in bone marrow hypocellularity and HSC loss [1]. Thus, the modest impact of *Men1* loss alone or menin/MLL1 disruption in normal hematopoiesis may result from compensatory activity of the TrxG gene *Ash1l*. It is also possible that individual molecular pathways differentially regulate recruitment of MLL1 and MLL1 fusion proteins to target genes, opening a therapeutic window. Systematic studies of TrxG gene networks in normal and malignant hematopoiesis could point to the Achilles' heel of MLL1-driven leukemia.
